# From Bioresources to Thermal Insulation Materials: Synthesis and Properties of Two-Component Open-Cell Spray Polyurethane Foams Based on Bio-Polyols from Used Cooking Oil

**DOI:** 10.3390/ma16186139

**Published:** 2023-09-09

**Authors:** Krzysztof Polaczek, Maria Kurańska, Elżbieta Malewska, Małgorzata Czerwicka-Pach, Aleksander Prociak

**Affiliations:** 1 Department of Chemistry and Technology of Polymers, Cracow University of Technology, Warszawska 24, 31-155 Cracow, Poland; maria.kuranska@pk.edu.pl (M.K.); elzbieta.malewska@pk.edu.pl (E.M.);; 2 Faculty of Chemistry, University of Gdansk, Wita Stwosza 63, 80-308 Gdansk, Poland

**Keywords:** spray polyurethane foam, open-cell foam, bio-polyol, municipal waste, used cooking oil, thermal insulation

## Abstract

Open-cell spray polyurethane foams are widely used as highly efficient thermal insulation materials with vapor permeability and soundproofing properties. Unfortunately, for the production of commercial foams, mainly non-renewable petrochemical raw materials are used. The aim of this study was to determine the possibility of completely replacing petrochemical polyols (the main raw material used in the synthesis of polyurethanes, alongside isocyanates) with bio-polyols obtained from used cooking oils, classified as waste materials. The research consisted of three stages: the synthesis of bio-polyols, the development of polyurethane foam systems under laboratory conditions, and the testing of developed polyurethane spray systems under industrial conditions. The synthesis of the bio-polyols was carried out by using two different methods: a one-step transesterification process using triethanolamine and a two-step process of epoxidation and opening oxirane rings with diethylene glycol. The obtained bio-polyols were analyzed using gel chromatography and nuclear magnetic resonance spectroscopy. The developed polyurethane foam formulations included two types of fire retardants: halogenated tris(1-chloro-2-propyl) phosphate (TCPP) and halogen-free triethyl phosphate (TEP). In the formulations of polyurethane systems, reactive amine catalysts were employed, which become incorporated into the polymer matrix during foaming, significantly reducing their emission after application. The foams were manufactured on both a laboratory and industrial scale using high-pressure spray machines under conditions recommended by commercial system manufacturers: spray pressure 80–100 bar, component temperature 45–52 °C, and component volumetric ratio 1:1. The open-cell foams had apparent densities 14–21.5 kg/m^3^, thermal conductivity coefficients 35–38 mW/m∙K, closed-cell contents <5%, water vapor diffusion resistance factors (μ) <6, and limiting oxygen indexes 21.3–21.5%. The properties of the obtained foams were comparable to commercial materials. The developed polyurethane spray systems can be used as thermal insulation materials for insulating interior walls, attics, and ceilings.

## 1. Introduction

Two-component spray polyurethane foams (SPFs) are one of the most efficient thermal insulation and soundproofing materials used in the construction industry. The advantages of SPFs include fast application, very good adhesion to many surfaces, and the elimination of thermal bridges, providing a reduction in energy losses. As a product, SPF insulation does not attract mold, mildew or bacteria. A disadvantage of SPFs is that their application requires trained personnel to operate the spraying machines, use personal protective equipment, and maintain mechanical ventilation during application [1,2]. SPF characteristics for both building and non-building applications are specified by various standards: ISO 8873 [3]; ASTM Standards C1029 [4], D7425 [5]; ULC Standards S705.1 [6] and S712.1 [7], and European Standards EN-14315 [8].

SPFs can have either an open-cell (ocSPF) or a closed-cell (ccSPF) structure. Applications for ccSPFs include the hydro and thermal insulation of roofs, walls, and foundations. Commercial ccSPFs generally have apparent densities of 30–60 kg/m^3^, closed-cell contents higher than 90%, thermal conductivity coefficients up to 28 mW/m·K and water absorption lower than 2% [9]. Standard ocSPFs have apparent densities of 8–15 kg/m^3^, closed-cell contents lower than 20%, and thermal conductivity coefficients of 35–42 mW/m·K. The main application of ocSPFs is as thermal insulation for attics, interior walls and ceilings. The high vapor permeability of ocSPFs provides effective moisture control, preventing the formation of mold and mildew in insulated spaces [10].

One component of SPF systems consists of a polyol or a mixture of polyols and catalysts, surfactants, emulsifiers, foaming agents and flame retardants. The other component is an isocyanate—typically polymeric methylene diphenyl diisocyanate (pMDI) [11].

Standard polyols used in ccSPF systems include Mannich polyols (aromatic polyols with tertiary nitrogen content, which contributes to their high reactivity) and polyester polyols [12]. Commercial Mannich polyols typically have viscosities of 5000–30,000 mPa∙s at 25 °C, functionalities of 4–5 and high hydroxyl values exceeding 400 mgKOH/g [13,14]. Polyester polyols typically have viscosities in the range of 600–6500 mPa∙s at 25 °C, functionalities of approximately 2–3 and hydroxyl values of 240–360 mgKOH/g [15,16].

For the production of ocSPFs, polyether polyols are commonly used due to their resistance to hydrolysis in high-water formulations (where water acts as the foaming agent) [17]. Polyether polyols generally have viscosities up to 10,000 mPa∙s, functionalities of 4–5 and hydroxyl values of 400–500 mgKOH/g [18,19]. OcSPF formulations can also contain amine-based polyols with hydroxyl values greater than 500 mgKOH/g and very high reactivity, which reduces the cream time (time at which the system starts to expand) [12,20].

The global polyols market in 2019 was worth USD 26 billion, and analysis [21] suggests there will be further growth due to the increasing production of polyurethanes. Commercial polyols are produced primarily from compounds of non-renewable origin, i.e., crude oil and natural gas. Both academic institutions [22] and polyol manufacturers [21] are intensively developing new bio-based polyols (bio-polyols) derived from renewable and waste materials to enhance the sustainability of the polyurethane industry. Commercial production of bio-polyols most often involves the use of fresh vegetable oils [23]. Raw materials that can also serve as a source for the synthesis of bio-polyols are: used cooking oils (UCOs) [24], oils from microalgae [25] and wastes from the cellulose industry i.e., lignin [26] or tall oil fatty acids [27]. UCO, thanks to its composition comparable to refined vegetable oil, is regarded as a significant and economically viable bioresource, primarily due to its low cost and widespread availability. It can also be easily recycled and repurposed for various applications, making it a renewable and sustainable resource, for the synthesis of polyvinyl chloride plasticizers [28] and epoxy resin components [29], as well as lubricants, asphalt additives, and fuels [30].

The most commonly used industrial methods for the synthesis of bio-polyols from vegetable oils are the two-step epoxidation and ring-opening process and the one-step transtesterification process [21].

Commercial SPFs containing bio-polyols are products with limited distribution. An example of such a product is Agribalance^®^ [31], which has an apparent density ranging from 9.6 to 12.8 kg/m^3^. According to the manufacturer’s information, 20% of its content constitutes renewable agricultural-based materials. The ccSPF system containing bio-polyols from soybean oil is HEATLOK^®^ SOYA HFO™ [32], which has an apparent density of about 35.5 kg/m^3^ and contains 4% renewable and 18% recycled materials.

The scientific literature on SPFs is very limited and mainly focuses on the development of closed-cell foams. Yakushin et al. [33] conducted research on the use of bio-polyols from rapeseed and sunflower oil obtained by transamidation to produce ccSPFs with reduced flammability and apparent densities of 34–38 kg/m^3^. The ratio of bio-polyol to petrochemical polyol was 70:30, and the produced foams were characterized by Class E fire response, similar to most commercially available ccSPFs. In another study [34], Yakushin et al. developed ccSPFs using a fourth-generation blowing agent with an ozone depletion potential (ODP) of about zero and a GWP (global warming potential) of 1, using only petrochemical polyols. Stirna et al. [35] obtained rigid polyurethane spray-applied coatings from rapeseed oil polyols such as rapeseed oil diethanolamides, rapeseed oil triethanolamine esters, and rapeseed oil monoglyceride. The coating produced using triethanolamine polyol had the most favorable properties.

The lack of research is particularly evident in the preparation of open-cell foams. Information on the manufacture of ocSPFs with densities <20 kg/m^3^ is available only in the patent literature [11,36,37]. The authors are aware of several papers [38,39,40,41] describing the synthesis of open-cell PUR foams with apparent densities from 25 to 90 kg/m^3^. However, these papers did not focus on the thermal insulating properties of the foams, and spray methods were not used for their manufacture.

A previous study [42] identified the most favorable parameters for the synthesis of bio-polyols from used cooking oil intended for ocSPF systems of low apparent density. In the presented study, ocSPF systems containing 100% polyol derived from a waste raw material (used cooking oil) were developed through the epoxidation/ring-opening and transesterification methods. Spray tests were conducted using an industrial machine under conditions recommended by ocSPF manufacturers. The main objective of the study was to compare the properties of open-cell foams with low apparent density produced in the laboratory and with spray equipment, as well as to investigate the effect of spray conditions, specifically the temperature and spray pressure.

## 2. Materials and Methods

### 2.1. Materials

Used cooking oil (UCO) with a viscosity of 68 mPa∙s at 25 °C, an acid value of 0.93 mgKOH/g, and an iodine value of 107 gI_2_/100 g (equivalent to 0.42 mol of double bonds per 100 g of UCO) was collected from a local restaurant in Cracow, Poland. The UCO contained small amounts of solid impurities remaining from the frying process, which were separated, first using a 40-mesh sieve filter, and then through decantation. The iodine value of the UCO used indicates that it mainly consisted of used rapeseed oil. The following materials were used for the synthesis of bio-polyols using a two-step epoxidation and oxirane ring-opening process: glacial acetic acid (99.5 wt.%), hydrogen peroxide (30 wt.%) and diethylene glycol (DEG) supplied by Avantor Performance Materials Poland S.A.(Gliwice, Poland); and ion exchange resin Amberlite^®^ IRC120 H and tetrafluoroboric acid (48 wt.% solution in water) supplied by Merck (Darmstadt, Germany). For the transesterification reaction of the UCO, triethanolamine (TEA) with a viscosity of 600 mPa⋅s at 25 °C and zinc acetate (catalyst), supplied by Chempur (Piekary Śląskie, Poland), were used. The catalysts, surfactantsand additives used for the development of ocSPF systems were provided by Evonik Industries AG (Essen, Germany). They included POLYCAT^®^15, Evonic Industries AG Essen, Germany (chemical name: Bis[3-(dimethylamino)propyl]amine; non-emissive balanced amine catalyst with slight selectivity towards the blowing reaction; calculated hydroxyl value of 282 mgKOH/g), POLYCAT^®^140 (chemical name: 2-(2-Aminoethoxy)ethanol; non-emissive amine catalyst with high selectivity towards the blowing reaction; calculated hydroxyl value of 413 mgKOH/g), and POLYCAT^®^142 (chemical name: 1,1,3,3-Tetramethylguanidine; non-emissive, highly efficient reactive amine promoting the blowing reaction with a quick initiation; calculated hydroxyl value of 435 mgKOH/g). The chemical structures of the used catalysts are presented in Table 1. The surfactants applied were TEGOSTAB^®^ B 8870 (polyether-polysiloxane-copolymer, strong stabilizer used in high-water formulations; calculated hydroxyl value of 60 mgKOH/g), TEGOSTAB^®^ B 8526 (polyether-polydimethylsiloxane-coplymer, cell-opener with some stabilizing properties; calculated hydroxyl value of 100 mgKOH/g), TEGOSTAB^®^ B 8523 (polyether-polydimethylsiloxan-coplymer,strong cell-opener; calculated hydroxyl value of 120 mgKOH/g), and ORTEGOL^®^ 500 (strong silicone-free cell-opener; calculated hydroxyl value of <5 mgKOH/g). Flame retardants tris(2-chloroisopropyl)phosphate (TCPP) with a viscosity of 67 mPa∙s at 25 °C and triethyl phosphate (TEP) with a viscosity of 1.7 mPa∙s at 25 °C were supplied by Purinova (Poland). The isocyanate pMDI Purocyn B with an isocyanate group content of 30–32%, density of 1.24 g/cm^3^ at 25 °C and viscosity within the range of 170–230 mPa∙s at 25 °C, were supplied by Purinova (Bydgoszcz, Poland). Distilled water played the role of a chemical blowing agent.

### 2.2. Synthesis of Epoxidized UCO

The epoxidized oil synthesis was carried out in a 10 dm^3^ reactor equipped with a heating–cooling mantle, a reflux condenser, a thermometer, and a mechanical stirrer. For the reaction, 6 kg of UCO, 2.4 kg of hydrogen peroxide, 0.3 kg of glacial acetic acid, and 1.2 kg of Amberlite^®^ IRC120 H were added to the reactor. The reaction was carried out at 65 °C for 6.5 h. The reaction mixture was separated and washed four times with 3 dm^3^ of warm water and distilled under reduced pressure. The epoxy value of the epoxidized UCO was 0.22 mol/100 g. Detailed information and a comprehensive description of the epoxidized oil and the bio-polyol synthesis method were previously presented in a study [42].

### 2.3. Synthesis of UCO-Based Bio-Polyol via the Epoxidation/Oxirane Ring-Opening Method

The synthesis of the bio-polyol was carried out in a 6 dm^3^ reactor equipped with a mechanical stirrer and a thermometer. A mixture of 4 kg of the epoxidized UCO was added to the reactor and heated up to 80 °C. Next, 16 g of a tetrafluoroboric acid solution and 0.94 kg of DEG were added to the reactor. The reaction was carried out for 60 min at 95–100 °C. The bio-polyol had a light-brown color and a delicate smell. The abbreviation for the UCO-based polyol obtained via the epoxidation/oxirane ring-opening method is BP_DEG.

### 2.4. Synthesis of UCO-Based Bio-Polyol (BP_TEA) Using the Transesterification Reaction

The selection of the reaction conditions, the catalyst concentration, and the type of transesterification agent were described in a previous study [24]. The reaction was carried out in a 6 dm3 reactor equipped with a magnetic stirrer, at atmospheric pressure under reflux conditions, and in an inert gas (nitrogen) atmosphere. For the reaction, 3 kg of UCO was added to the reactor and heated to 175 °C. Then, 1475 g of TEA (the molar ratio of the transesterification agent to UCO was 3:1) and 9 g of zinc acetate (0.45 wt.% related to the UCO mass) were added to the reactor. The reaction was conducted at 175 °C for 2 h. The bio-polyol had a dark-brown color. The presence of a tertiary amine in the bio-polyol’s structure causes its built-in catalytic activity [43], as well as its ability to reduce the flammability of the PUR foam [44]. The abbreviation for the UCO-based polyol obtained via transesterification reaction is BP_TEA.

### 2.5. Development of ocSPF Systems at Laboratory Scale

In the first stage of the study, the formulations of ocSPFs were developed based on previous research [42] by conducting laboratory-scale syntheses. Open-cell polyurethane foams were prepared using a one-step method with a two-component system. Component A consisted of a bio-polyol, catalyst, surfactant, flame retardant, and water, while Component B was isocyanate. After placing all the raw materials into the container, Component A was mixed for approximately 30 s at a stirring speed of about 1500–2000 rpm to achieve thorough homogenization. The volume ratio of Component A to Component B was 1:1, and the components were maintained at a temperature of 45 °C to simulate standard polyurethane foam production conditions using a high-pressure spray machine. After reaching the desired temperature, Component B was rapidly poured into the container containing Component A, and the mixture was stirred for approximately 3 s before being poured into an open mold. The obtained foams were removed from the container 24 h after pouring, and samples were cut out for testing.

The developed ocSPFs contained only one type of bio-polyol. The formulations of ocSPF systems are detailed in Table 2. The foams produced under laboratory conditions with BP_DEG were designated as DEG_t45_lab, and the foams containing the BP_TEA bio-polyol were designated as TEA_t45_lab. For the DEG_t45_lab formulation, the substitution of TEP by TCPP was necessary to achieve dimensionally stable foams with homogeneous cell structures.

The isocyanate index is a critical parameter in the production of polyurethane materials. It represents the ratio of the actual isocyanate content (NCO groups) in a polyurethane formulation to the theoretical or stoichiometric isocyanate content required for a complete reaction with the other components. By adjusting the isocyanate index, the properties of the final polyurethane product, such as its hardness, flexibility, and other mechanical characteristics, can be modified. The application of the polyurethane spray system using a high-pressure machine requires an equal-volume ratio of Component A to Component B, which affects the isocyanate index of the polyurethane system. A higher isocyanate index, leading to a greater degree of crosslinking in the polyurethane material, typically results in a more rigid and harder polyurethane, while a lower index produces a softer and more flexible material.

The isocyanate index was calculated as the ratio of the number of moles of isocyanate groups to the number of moles of hydroxyl groups and other groups capable of reacting with isocyanate groups.

### 2.6. Manufacturing ocSPF Using a High-Pressure Spray Machine

**Figure 1:**
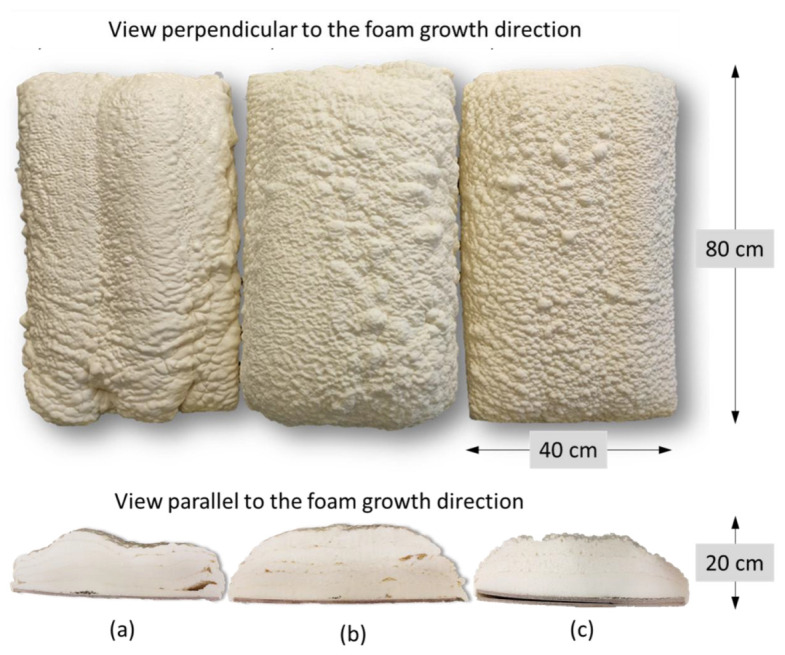
Sprayed ocSPF containing BP_DEG: (**a**) DEG_p80_t52; (**b**) DEG_p90_t52; (**c**) DEG_p100_t52.

**Figure 2:**
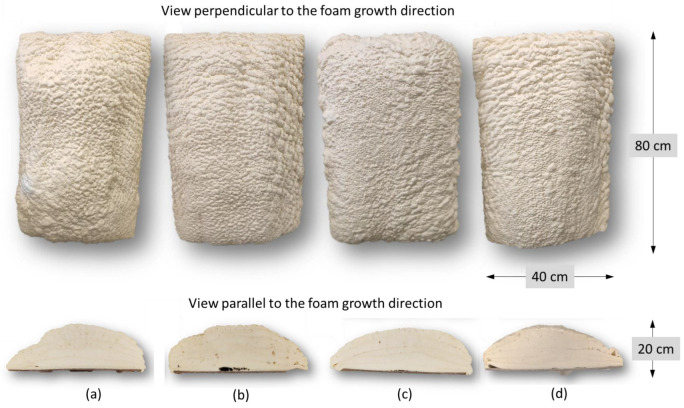
Sprayed ocSPF foams containing BP_TEA: (**a**) TEA_p80_t45; (**b**) TEA_p80_t52; (**c**) TEA_p90_t52; (**d**) TEA_p100_t52.

The developed two-component ocSPFs were obtained by using a Reactor E-20 machine made by Graco Inc. (Minneapolis, MN, USA) equipped with a Graco Fusion^®^ AP spray gun. Spray conditions were selected according to ocSPF manufacturers’ recommendations. The temperature of the components at the mixing head inlet was 45 and 52 °C in the case of TEA_ocSPF and 52 °C in the case of DEG_ocSPF, due to its high viscosity in lower temperatures. The viscosity of polyol premixes is shown in Table 3. The addition of flame retardants, catalysts, and water significantly affects the viscosity of polyol premixes, which are lower than those of bio-polyols (Table 4). The spray pressures were 80, 90, and 100 bar for both ocSPF systems. The ambient and sprayed surface temperature was 17–19 °C. Spraying was carried out on horizontally placed cardboard. Component A was a bio-polyol, catalyst or catalyst mixture, surfactants, flame retardant, and water. Component B was an isocyanate pMDI. The volume ratio of the components was 1:1. The spray system naming pattern is as follows: [bio-polyol_spray pressure_temperature of components]. Pictures of the foams and their cross-sections are shown in Figure 1 and Figure 2.

Spray pressure affects the homogenization quality of the polyurethane system components. Visually, it was found that increasing the spray pressure resulted in the formation of fewer voids in the foam and between subsequent sprayed layers (Figure 1 and Figure 2). In the context of the ocSPF containing BP_DEG, the foam characterized by the most favorable structure was obtained at a spray pressure of 100 bar, denoted as DEG_p100_t52. Spray pressures of 80 and 90 bar were found to potentially impede effective component homogenization, consequently leading to a deterioration of the foam’s structure. In the case of ocSPF systems containing BP_TEA, the presence of a significant number of voids and spaces was exclusively observed in the TEA_p80_t52 foam (Figure 2b).

### 2.7. Characterization

#### 2.7.1. Raw Materials Analysis

The hydroxyl values (Hv) and the epoxy values (Ev) of the polyols were found on the basis of the PN-93/C-89052/03 [45] and PN-87/C-89085/13 standards [46], respectively.

The number-average molecular weight (Mn), weight-average molecular weight (Mw), and dispersity (D) of the compounds used in the experiments were determined using a gel permeation chromatograph made by Knauer (Berlin, Germany). The device was equipped with thermostatic columns and a refractometric detector. The analyses were performed at 25 °C. Tetrahydrofuran was used as an eluent and its flow rate was fixed at 1 mL/min.

The water content was found using Karl Fischer’s method according to the PN-81/C-04959 standard [47] with the use of a TitroLine KF device manufactured by SI Analytics GmbH (Mainz, Germany).

The viscosity was determined with a HAAKE MARS III rotary rheometer from Thermo Scientific (Waltham, MA, USA). A plate-to-plate system at 100 rpm was used. The test was conducted at 25 and 45 °C.

Functionality (f) was calculated based on Formula (1):

(1) $$f=\frac{M_{n}\cdot H_{v}}{56,100}$$

where *M_n_* is the number-average molecular weight [mol/g]; *H_v_* is the hydroxyl value of the polyol [mg KOH/g].

Nuclear magnetic resonance (NMR) 1D experiments (^1^H NMR) were recorded with a Bruker DRX 500 Avance 500 MHz spectrometer using the standard Bruker software (TopSpin 3.1) and deuterated chloroform (CDCl_3_) as a solvent. Chemical shifts were reported relative to the 1H NMR chloroform signals at δ = 7.26 parts per million (ppm).

#### 2.7.2. Measurement of Polyurethane Foams Properties

The closed-cell content was studied according to the ISO 4590 standard [48]. The test samples were 25 × 25 × 100 mm in size.

The heat conduction coefficient (λ) of the PUR foams was determined using a LaserComp FOX 200 apparatus from TA Instruments (New Castle, DE, USA). All samples had a size of 200 × 200 × 50 mm. The temperature difference between the hot and cold plate was 20 °C.

The compressive strength test was carried out in accordance with the ISO 844 standard [49] using a Zwick Z005 TH testing machine (Zwick GmbH & Co, Ulm, Germany). Samples of cylindrical shape with a diameter of 40 mm and a height of 40 mm were cut out from the foam cores. The compressive strength was determined parallel to the direction of foam growth at 10% deformation.

The apparent densities of the foams were determined on the basis of the measurements of the samples masses and volumes based on the ISO 845 standard [50]. Foam samples with a size of approximately 200 × 200 × 50 mm were used.

The brittleness test was carried out according to the ASTM C 421-08 standard [51] and the result is presented as the percentage weight loss of the sample.

The water-vapor permeability (δ) and the water-vapor diffusion resistance factor (μ) were determined according to the PN-EN 12086:2013 standard [52]. The test specimen (polyurethane foam) with a diameter of 50 mm and thickness of 20 mm was sealed to the open side of a cylindrical test dish containing a desiccant–anhydrous calcium chloride. In the test, the temperature was 23 °C and relative humidity was 85%. Six samples from each foam were tested. The weight of the test assembly was measured at regular 24 h intervals until the change in mass was constant within ±5% of the mean value.

The limiting oxygen index (LOI) test was performed on the basis of the ISO 4589-2 standard [53].

The foam morphology was examined using a HITACHI S-4700 (Hitachi, Ltd., Tokyo, Japan) scanning electron microscope (SEM) (configured with a secondary electron detector in low vacuum mode and an acceleration voltage of 20 kV. Magnifications of 35 times were used. Analysis of the foams’ morphology (number of cells, cell cross-section area and anisotropy index) was performed using ImageJ software ver. 1.53 (U.S. National Institutes of Health, Bethesda, MD, USA, https://imagej.nih.gov/ij/, accessed on 5 September 2023, t). The cellular density of foams was calculated using the following Equation (2), which is adequate for the analysis of foams with cells elongated in the flow direction.

(2) $$N=N_{PA}{\left(N_{PE}\right)}^{1/2}$$

where *N* is the cellular density expressed as the number of cells in a cm^3^, *N_PA_* is the number of cells in direction parallel to the foam growth direction in the area of the image cross-section, *N_PE_* is the number of cells in directions perpendicular to the foam growth direction in the area of the image cross-section.

## 3. Results and Discussion

### 3.1. Bio-Polyol Synthesis

At the first stage of the study, bio-polyols from UCO using two methods were synthesized. The basic properties of the UCO and bio-polyol produced are shown in Table 4.

**Figure 3:**
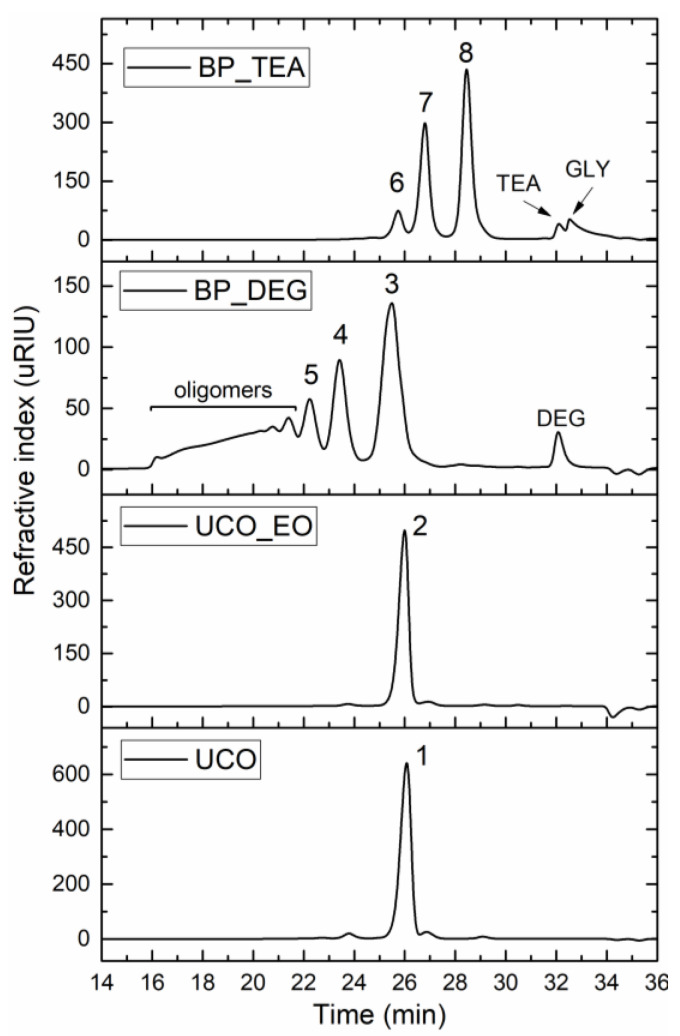
GPC chromatograms of UCO, UCO_EO and bio-polyols obtained by two processes.

UCO_EO was characterized by a slightly increased Av, molecular weight, viscosity, and water content compared to UCO. The BP_TEA bio-polyol had a higher hydroxyl number, but lower viscosity and functionality compared to the BP_DEG bio-polyol. Both polyols had higher viscosity than the initial UCO. Samples of the UCO, UCO_EO and the bio-polyols were analyzed by GPC chromatography, and the results are presented in Figure 3.

The GPC analysis of UCO revealed the presence of triglyceride (peak 1 with a retention time of 26 min), and small amounts (approximately 2.5% by mass) of compounds formed during the previous high-temperature usage of the vegetable oil [54]. The GPC analysis of UCO_EO (peak 2 with a retention time of about 26 min) showed a slight increase in the molecular weight of triglycerides, primarily due to the addition of oxygen atoms. During the epoxidation stage, besides the main reaction, side reactions can also occur, leading to an increase in the molecular weight of triglycerides. Typical side reactions of epoxidation reactions using organic peracids include reactions that result in the formation of secondary hydroxyl groups through the opening of oxirane rings by water (hydrolysis), homopolymerization, or acylation to form hydroxyesters [55]. The GPC analysis did not conclusively indicate an increased number of dimers or other side compounds in the UCO_EO sample compared to the UCO sample.

**Figure 4:**
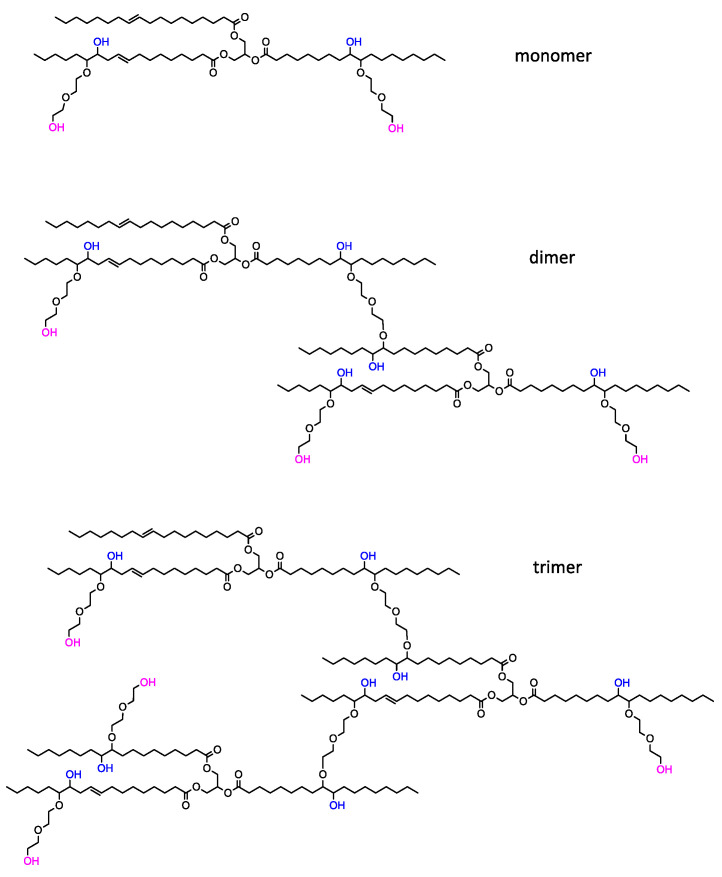
Idealized chemical structure of bio-polyol BP_DEG formed solely in the hydroxylation reaction of UCO_EO and DEG, along with the by-products of dimerization and oligomerization reactions. Purple indicates primary OH groups; blue indicates secondary OH groups.

**Figure 5:**
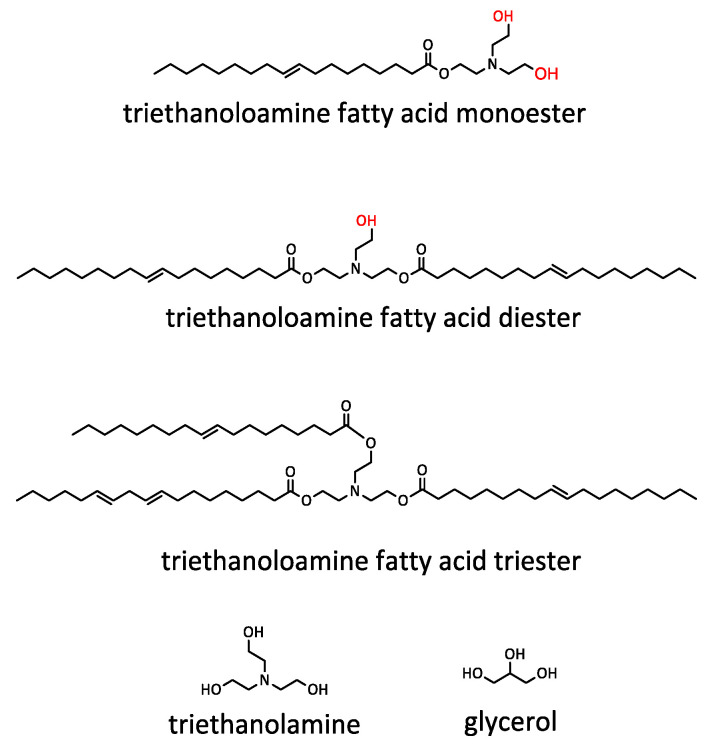
Idealized chemical structure of BP_TEA mixture obtained by transesterification reaction. Red indicates primary OH groups.

The BP-DEG bio-polyol obtained through epoxidation and oxirane ring-opening is a product of a heterogeneous composition. Peak 3, with a retention time of 25.5 min, corresponds to hydroxyl derivatives of triglycerides (monomers). Peaks 4 and 5, with retention times of 23.7 min and 22.2 min, respectively, originate from dimers and trimers. The remaining peaks with retention times ranging from 21.7 min to 16.1 min indicate the presence of oligomers. High-molecular-weight compounds, formed inside reactions involving the opening of an oxirane group by hydroxyl groups previously incorporated into the triglyceride structure, are responsible for the high viscosity of the BP_DEG bio-polyol. Previous studies have shown that the intensity of side reactions increases with an increase in the epoxy group content [42]. The bio-polyol BP_TEA obtained in the UCO transesterification reaction exhibited a different chemical structure than the bio-polyol BP_DEG. The BP_TEA bio-polyol contained small amounts of triethanolamine fatty acid triesters (peak 8 with a retention time of 25.7 min), triethanolamine fatty acid diesters (peak 7 with a retention time of 26.8 min), triethanolamine fatty acid monoesters (peak 6 with a retention time of 28.5 min), and unreacted TEA and glycerol (GLY) formed during the transesterification reaction. The GPC analysis and the peak separation quality did not give a clear result regarding the presence and content of unreacted glycerol esters in the BP_TEA bio-polyol, whose peaks, as a result of small differences in molecular weight with respect to triethanolamine esters, may overlap with peaks 6, 7 and 8. The high content of diesters and monoesters is responsible for the low viscosity of the BP_TEA bio-polyol. Hypothetical chemical compositions of bio-polyols BP_DEG and BP_TEA are shown in Figure 4 and Figure 5, respectively.

The reaction of oxirane ring-opening by DEG leads to the formation of bio-polyols containing both primary and secondary OH groups (Figure 4). Primary OH groups are characterized by higher reactivity in the reaction with isocyanates compared to secondary OH groups [56]. The bio-polyol BP_TEA mainly consists of short-chain triethanolamine fatty acid esters of functionality 2 (Figure 5), where both hydroxyl groups are primary.

**Figure 6:**
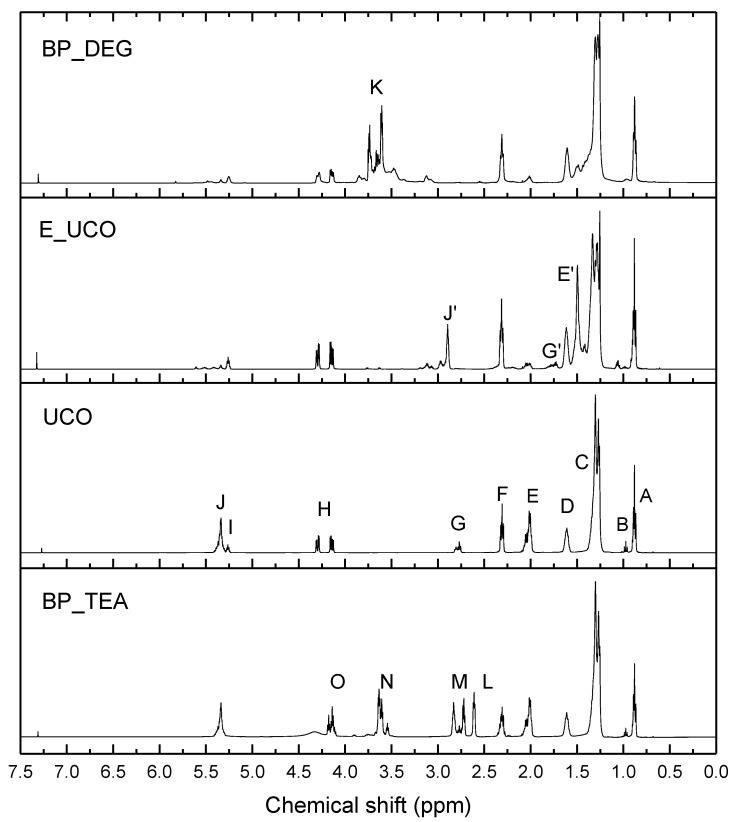
^1^H NMR spectrum of UCO, E_UCO, BP_TEA and BP_DEG.

The ^1^H NMR spectrum (Figure 6) of the used cooking oil (UCO) contained several typical signals of major groups of culinary oils: (A) δ (chemical shift) 0.87–0.90 ppm(–CH_2_–C**H**_3_); (B) δ 0.96–0.99 ppm (=CH–CH_2_–C**H**_3_); (C) δ 1.26–1.37 ppm (–C**H**_2_–); (D) δ 1.61 ppm (β–CH_2_ group of the carbonyl group –C**H**_2_–CH_2_–CO–); (E) δ 1.99–2.07 ppm (–CH_2_–C**H**_2_–CH=CH–); (F) δ 2.29–2.33 ppm (α-CH**_2_** group of the carbonyl group –CH_2_–C**H**_2_–CO–); (G) δ 2.76–2.82 ppm (–CH=CH–C**H**_2_–CH=CH–); (H) δ 4.13–4.30 ppm (methylene protons of glyceryl –C**H**_2_–CH–C**H**_2_–); (I) δ 5.26 ppm (methine protons of glyceryl –CH_2_–C**H**–CH_2_–); (J) 5.34 ppm (protons of olefin groups of fatty acids –C**H**=C**H**–). These results are in good agreement with the literature data [57]. The ^1^H NMR spectra of the epoxidized UCO (E_UCO) and the UCO-based bio-polyol obtained via the epoxidation/oxirane ring-opening method (BP_DEG) revealed the disappearance of unsaturated double bonds and confirmed the proceeding reactions of epoxidation and oxirane ring-opening (Figure 4). The signals (B) (=CH–CH_2_–C**H**_3_), (E) (–CH_2_–C**H**_2_–CH=CH–), (G) (–CH=CH–C**H**_2_–CH=CH–) and (J) (–C**H**=C**H**–) became weaker. Instead, new signals appeared in the case of the proton NMR spectrum of E_UCO: (J’) δ 2.94–3.04 ppm (–**H**C–O–C**H**– epoxy ring); (E’) δ 1.54 ppm (–HC–O–CH–C**H**_2_–CH_2_); (G’) δ 1.76–1.86 ppm (–C**H**_2_– adjacent to epoxy groups). In the spectrum of BP_DEG, several new signals were observed in the area (K), indicating the incorporation of DEG into the bio-polyol structure (newly created methylene groups and hydroxyl groups), and there was a lack of signals—(J’), (E’) and (G’)—characteristic of epoxy groups. Similar observations concerning the NMR data of the epoxidized oils have already been made by other researchers [29,58,59].

The BP_TEA bio-polyol was a major product obtained by the transesterification reaction (Figure 5). It was evidenced by the presence of signals (L) δ 2.56 (–N(**CH_2_**–CH_2_–OH)_2_), (M) δ 2.67 (–O–CH_2_–**CH_2_**–N(CH_2_–CH_2_–OH)_2_), (N) δ 3.43–3.62 (–N(CH_2_–**CH_2_**–OH)_2_), (O) δ 4.0–4.15 (–O–**CH_2_**–CH_2_–N(CH_2_–CH_2_–OH)_2_). Signals of protons (HO–**CH_2_**–**CH**(OH)–**CH_2_**–OH) from glycerol were also observed in the region (N). Other signals correspond to protons typical of UCO fatty acid chains but also methylene and methine groups from the rest of the products obtained after the transesterification reaction (different mono- and diesters).

### 3.2. Polyurethane Foam Synthesis on Laboratory Scale

The development of spray foam formulations requires the selection of an appropriate catalytic system that provides uniform foam growth. The catalytic mechanisms described in the literature for PUR formation follow two potential pathways: increasing the electrophilic character of the carbon in the isocyanate group or increasing the nucleophilic character of the compound with labile hydrogen atoms. Catalysts used in PUR formulations are categorized into two groups: metal catalysts promoting the polyol-isocyanate reaction (gelation reaction), resulting in the formation of urethane bonds, and amine catalysts promoting mainly isocyanate–water reactions (foaming reactions), resulting in the formation of unstable carbamic acid, which immediately decomposes into carbon dioxide and amine [60]. Balanced amine catalysts promoting both gelling and foaming reactions are the most commonly used in ocSPF systems. Manufacturers of spray polyurethane foams generally use conventional, emissive catalysts due to their high reactivity. Modern ocSPF amine catalysts belong to a group of reactive catalysts, building into the structure of a polymeric matrix [61]. Compared to commonly used conventional non-reactive foaming catalysts, such as bis-(2-dimethylaminoethyl)ether (BDMAEE), the use of reactive catalysts significantly reduces off-gassing over time, fishy odor after foaming, and the potential for skin, eye or respiratory system irritation [1,62,63]. The disadvantage of reactive catalysts is their decreasing catalytic activity, associated with loss of mobility after incorporation into the structure of the polymer network [60]. The incorporation of reactive catalysts into the polymer structure occurs due to the reaction of functional groups present in tertiary amines, which are able to react with isocyanate. This results in covalent bonding, preventing the release of the amine into the environment. However, the incorporation of a reactive amine can act as a chain terminator, thus hindering polymer chain growth and leading to the deterioration of foam properties. An important drawback of reactive amine catalysts is the need to use them in larger quantities compared to conventional catalysts, due to their irreversible immobilization.

**Figure 7:**
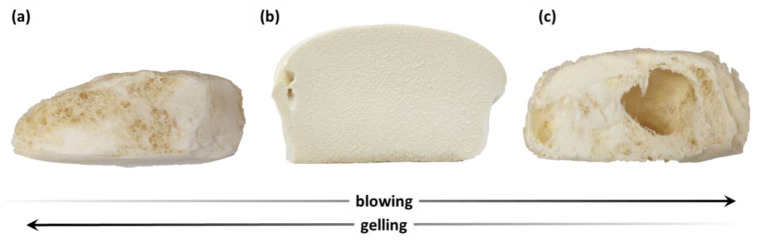
Effect of the addition of gelling and blowing catalysts on the structure of open-cell foams: (**a**) polycat 140—9 pbw, polycat 15—3.5 pbw; (**b**) polycat 140—12 pbw, polycat 15—3.5 pbw; (**c**) polycat 140—12 pbw, polycat 15—1.5 pbw.

The development of open-cell polyurethane foam systems containing only reactive amine catalysts required the selection of optimal catalysts and their quantities. Figure 7 shows the effect of the gelling and blowing reactive catalysts on the cell structure of the open-cell foams obtained in the research.

The reactive amine catalysts used in the ocSPF formulations had both gelling and blowing (foaming) properties, and their chemical structure determined which way the selectivity was shifted. An excessive selectivity towards gelling or insufficient foaming resulted in foams with high apparent density and an improper cell structure (Figure 7a). Rapid blowing reactions combined with insufficient gelling reactions resulted in the formation of a foam with a fibrous structure containing numerous holes and cavities (Figure 7c). The correct foam structure is presented in Figure 7b. The effect of catalysts on the structure of polyurethane foams has been well described in the literature [60]. In general, when carbon dioxide is released too intensively and the viscosity of the reaction mixture is too low, the emulsion is destabilized, and large cells are formed. On the contrary, when the gelling reaction occurs too intensely compared to the blowing reaction, the viscosity of the mixture increases rapidly, and the cells do not expand to their optimal volume.

**Figure 8:**
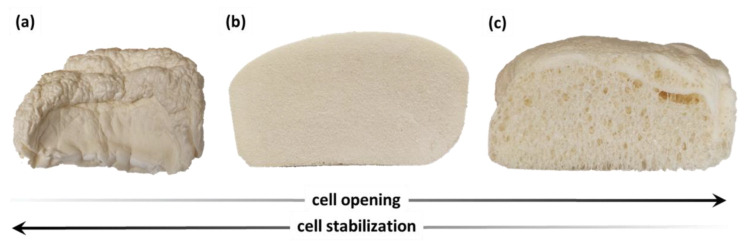
Effect of the addition of cell-stabilizing surfactant and cell-opener on the structure of open-cell foams: (**a**) TEGOSTAB 8870—1.2 pbw, TEGOSTAB 8526—0 pbw; (**b**) TEGOSTAB 8870—1.2 pbw, TEGOSTAB 8526—0.6 pbw; (**c**) TEGOSTAB 8870—1.2 pbw, TEGOSTAB 8526—0.9 pbw.

The homogeneous cell structure of polyurethane foam cannot be obtained without the use of surfactants. The major roles of surfactants are to emulsify the reactants and to stabilize the growing cells [60]. The ocSPF systems make use of surfactants with both cell-stabilizing and cell-opening properties. The foaming process of open-cell foams can be divided into the following stages: bubble nucleation and growth, packing of the bubble network and cell stabilization, polymer network stiffing, and cell opening and final solidification [64]. The proper selection of ocSPF surfactants involves finding a balance between cell stabilization and cell opening. If surfactants with excessive stabilization (or insufficient cell opening) properties are used, small, closed cells that tend to shrink may form. On the other hand, poor stabilization results in the formation of cells with excessively large sizes, which adversely affects the foam’s thermal insulation and mechanical properties. Using an excessive amount of cell-openers may lead to a complete collapse of the foam [61]. Figure 8 shows the effect of an addition of stabilizing and cell-opening surfactants on the foams obtained in the research.

The addition of either a too-high amount of the cell-stabilizing surfactant or a too-low amount of the cell-opening surfactant resulted in the formation of foams with closed cells of small sizes, which increased the tendency of the foam to shrink (Figure 8a). The addition of either an excessive amount of a cell-opener or an insufficient amount of the stabilizing surfactant resulted in the formation of foam with large cell sizes (Figure 8c). The proper foam structure is depicted in Figure 8b.

Table 5 presents selected properties of the PUR foams with the most favorable properties obtained on a laboratory scale.

The polyurethane foams obtained in the laboratory were mixed using a mechanical mixer, which influenced their cell structure but provided a preliminary assessment of their feasibility for larger-scale testing. Both foams were characterized by low apparent densities and very low closed-cell contents. Incorporating the flame-retardant properties of BP_TEA allowed for a reduction in the foam’s flame-retardant content by 10 g/100 g of polyol (Table 2) while maintaining a higher LOI of 21.5% compared to the foam based on BP_DEG bio-polyol (21.3%). The high brittleness of the TEA_t45_lab foam is attributed to the chemical structure (high hydroxyl value and low molecular weight) of the BP_TEA bio-polyol, as described in detail in Section 3.3.

### 3.3. Polyurethane Foam Synthesis Using Industrial Spray Machine

Table 6 shows the selected properties of the foams obtained using a high-pressure spray machine under different spraying conditions.

The apparent density of the foams ranged from approximately 14 kg/m^3^ for DEG_p90_t52 and DEG_p100_t52 to over 21 kg/m^3^ for TEA_p80_45. All of the ocSPFs had higher apparent densities compared to the foams synthesized in the laboratory with the use of a mechanical mixer (Table 5). The apparent density of commercial ocSPF systems based on petrochemical polyols is typically from 7 to 9 kg/m^3^ [15,65,66] and from 9.6 to 12.8 kg/m^3^ for commercial ocSPF systems containing vegetable oil-based bio-polyols [31]. It was observed that increasing the temperature of spraying decreased the apparent density of the TEA foams. Investigating the effect of temperature on the DEG system was not possible due to its too-high viscosity at 45 °C. In the case of the DEG system, it was observed that increasing the spray pressure from 80 to 90 bar reduced the apparent density of the foams. No such correlation was observed for the TEA system.

All of the foams had closed-cell contents below 5%, which makes them dimensionally stable and shrinkage-free. In the case of the DEG system, a slight increase in the content of closed cells when increasing spray pressure was observed. For the TEA system, the closed-cell content was at a relatively constant level, decreasing only when sprayed at 100 bar (TEA_p100_t52).

The thermal conductivity coefficient of the foams is about 35 to 37 mW/m∙K, which is the standard value for foams with an open-cell structure. The DEG_p80_t52 foam with the lowest closed-cell content among all the foams had the highest thermal conductivity coefficient. TEA_p80_t45 with a higher closed-cell content (more than 4%) and the highest apparent density had the lowest thermal conductivity coefficient. Heat transfer in PUR foams occurs by natural convection, conduction of the gas phase inside the cells, conduction of solid polymer, and radiation [67,68]. In the case of open-cell foams, in which the gas can flow freely throughout the foam and is not trapped inside cells, the cell morphology, including cell diameter, anisotropy, wall, and strut thickness, has a significant impact on thermal conductivity. Generally, reducing the cell size limits the free convection of gases, leading to a reduction in heat transfer. Smaller cell sizes also result in a decrease in heat transfer by radiation. This is because cells with smaller diameters and thicker walls absorb and scatter a greater number of photons involved in heat transport by radiation. However, in the case of open-cell foams, heat transfer by radiation is practically negligible. In contrast, heat transfer through the solid phase increases as the apparent density of a PUR foam increases [67].

The flammability of the foams was tested by determining the limiting oxygen index (LOI). The DEG and TEA foams had an LOI of 21.3% and 21.5% for, respectively. An LOI of >21% indicates that under certain specific conditions, the ignited sample is self-extinguishing in an atmosphere (the concentration of oxygen in sea-level air is approximately 21%) [69].

The mechanical strength of polyurethane foams is highly dependent on their apparent density, cell structure, closed-cell content, and the crosslinking density of the polyurethane matrix. In the cases of the foams described here, the apparent density of the foams might have had the greatest influence on the results of the test. The foam with the highest apparent density, TEA_p80_t45, had the highest mechanical strength, and DEG_p90_t52 and DEG_p100_t52, with the lowest apparent densities, had the lowest mechanical strength. Commercial ocSPFs have a compressive strength at 10% strain ≥10 kPa, which indicates that with the exception of the DEG_p90_t52 foam, all the other foams boast parameters similar to those of the commercial products [9].

Very significant differences between the DEG and TEA bio-polyol-based foams were observed in terms of their brittleness. Polyurethanes have segmented structures composed of soft and hard segments. The soft segments are usually built from polyol chains, while the hard segments are composed of isocyanates and a chain extender [70]. Bio-polyol BP_DEG, with a high molecular weight, provides high elasticity of the polymer matrix, which results in the low brittleness of the BP_DEG-based foams. Bio-polyol BP_TEA has a low molecular weight and the position of hydroxyl groups is at the end of the chains (Figure 5), resulting in the higher brittleness of the foam.

A characteristic feature of open-cell foams is their high vapor permeability, which provides proper moisture removal from insulated areas, preventing the condensation of water vapor and the formation of mold and mildew. The results of the water-vapor permeability tests carried out as part of this research are presented in Table 7.

The vapor permeability of PUR foams is mostly influenced by the content of closed cells. As the content of closed cells increases, the water-vapor permeability decreases. The maximum value of the water-vapor diffusion resistance factor (μ) for the foam systems was approx. 5 (the less the better), which is within the standard range for commercial ocSPFs [9].

**Figure 9:**
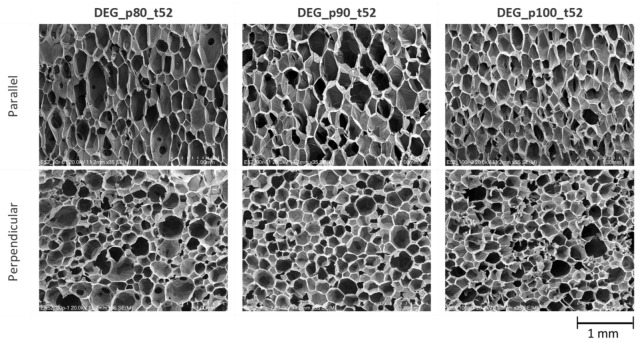
SEM microphotographs of DEG foams.

**Figure 10:**
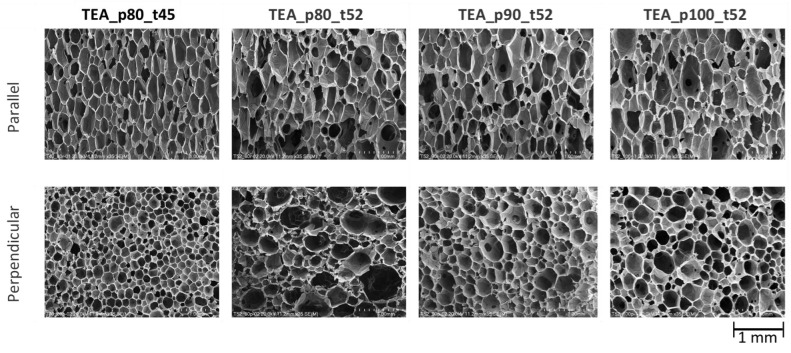
SEM microphotographs of TEA foams.

The obtained foams were analyzed in terms of their cellular morphology parallel and perpendicular to the growth direction (Table 8). Figure 9 shows SEM microphotographs of DEG foams. Figure 10 shows SEM microphotographs of TEA foams.

For DEG foams, a clear increase in cell density with increasing spray pressure was observed. For TEA foams, a similar tendency was not observed. One explanation may be the effect of the viscosity of the reaction mixture on the cellular structure, as reported in the literature [60]. During the foam growth, carbon dioxide is released, and, in the case of a low-viscosity mixture, the bubbles tend to coalesce, resulting in a foam with larger cells. When a higher-viscosity polyol is used, the concentration of growing bubbles is higher due to reduced diffusion and limited coalescence. In addition, the use of polyols with a branched structure increases nucleation, which leads to the formation of a larger number of small cells. The TEA bio-polyol used in the study has a much lower viscosity and a less-branched structure compared to the DEG bio-polyol. Foams produced with the TEA polyol are also characterized by a more elongated shape (larger anisotropy index) compared to foams based on the DEG polyol, which can also be explained by the different viscosity of the systems used.

A very large effect of spray temperature was observed on the cell density of the foam TEA_p80_t45. This foam had the highest cell density and the highest apparent density of all the foams produced. According to the literature, at a lower temperature, the polymerization reaction is slower, and more nucleation sites can form, leading to the formation of a large number of small cells, which increases the apparent density of the foam [71].

## 4. Conclusions

Two types of bio-polyols, derived from used cooking oil, were used as raw materials for the production of open-cell polyurethane spray foams, both in laboratory settings and industrial conditions, using a high-pressure spray machine. It was observed that the optimal spraying pressure for achieving favorable foam properties ranged from 90 to 100 bar. As the spray pressure increased, the apparent density of the foams decreased. Foams produced from bio-polyols obtained through a two-step process involving epoxidation and oxirane ring-opening with diethylene glycol were characterized by an apparent density in the range of 14 to 16 kg/m^3^. Foams based on bio-polyols obtained through a one-step transesterification process with triethanolamine had an apparent density within the range of 17 to 21.5 kg/m^3^. The bio-polyol obtained via transesterification with triethanolamine was characterized by flame-retardant properties, allowing for a 33% reduction in the amount of fire retardance compared to foams based on the bio-polyol obtained through epoxidation and oxirane ring-opening while simultaneously increasing the limiting oxygen index from 21.3% O_2_ to 21.5% O_2_.

All the obtained foams had a higher apparent density compared to commercial foams (typically not exceeding 10–12 kg/m^3^); however, they exhibited similar properties, such as heat conduction coefficient (34–37 mW/m∙K), mechanical strength (>10 kPa), closed-cell content (<5%), and water-vapor diffusion resistance factor (μ < 5). The manufactured polyurethane foam systems have the potential for use as thermal insulation materials to insulate interior walls, ceilings, and attics using high-pressure spray machines.

Further research is necessary, particularly to reduce the apparent density of these foams. Formulations incorporating both types of bio-polyols, whether obtained through epoxidation/ring-opening or transesterification, are worth future investigation. Moreover, the presented study underscores the variation in properties between polyurethane foams produced in laboratory settings and those manufactured under industrial conditions. This aspect should be carefully considered in future research focused on the production of such materials.

## Figures and Tables

**Table 1:** Chemical structure of the used catalysts.

Catalyst	Chemical Structure
POLYCAT^®^ 15	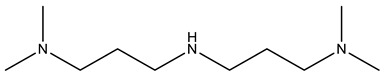
POLYCAT^®^ 140	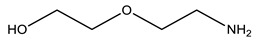
POLYCAT^®^ 142	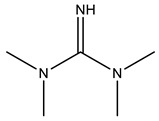

**Table 2:** Formulations of open-cell spray polyurethane foam systems based on different bio-polyols.

	BP_DEG-Based ocSPF		BP_TEA-Based ocSPF	
	Component	Share, pbw	Component	Share, pbw
Bio-polyol	BP_DEG	100	BP_TEA	100
Catalysts	POLYCAT^®^ 15	3.5	POLYCAT^®^ 142	5
	POLYCAT^®^ 140	12		
Surfactants	TEGOSTAB^®^ 8870	1.2	TEGOSTAB^®^ 8870	2
	TEGOSTAB^®^ 8526	0.6	TEGOSTAB^®^ 8523	0.6
	ORTEGOL^®^ 500	0.8	ORTEGOL^®^ 500	1
Blowing agent	Water	20	Water	20
Flame retardant	TCPP	30	TEP	20
Isocyanate	pMDI	208 *	pMDI	186 *
Isocyanate index	0.56		0.47	

* calculated according to a pMDI density of 1.24 g/cm^3^, at polyol premixes densities of 1 g/cm^3,^ and at a volume ratio of isocyanate to polyol premix of 1:1.

**Table 3:** Viscosity of polyol premixes.

Component A	η (25 °C), mPa∙s	η (45 °C), mPa∙s
DEG_ocSPF	1233 ± 18	366 ± 1
TEA_ocSPF	98 ± 1	47 ± 1

η—viscosity (mPa·s).

**Table 4:** Properties of UCO and bio-polyols.

Sample	Hv, mgKOH/g	Av, mgKOH/g	Mn, g/mol	Mw, g/mol	D	f	η (25 °C), mPa∙s	%H20, wt.%
UCO	-	0.93 ± 0.04	886	889	1.00	-	73 ± 1	0.10 ± 0.02
UCO_EO	-	1.78 ± 0,03	918	926	1.01	-	127 ± 2	0.24 ± 0.03
BP_TEA	349 ± 3	2.31 ± 0.12	340	553	1.62	2.1	226 ± 2	0.31 ± 0.04
BP_DEG	214 ± 2	2.92 ± 0.08	1874	4307	2.29	4.8	3384 ± 5	0.29 ± 0.03

Hv—hydroxyl value (mg KOH/g); Av—acid value (mg KOH/g); Mn—number-average molecular weight (g/mol); Mw—weight-average molecular weight (g/mol); D—dispersity, f—functionality; η—viscosity (mPa·s); % H_2_O—content of water (wt.%).

**Table 5:** Selected properties of open-cell PUR foams obtained on a laboratory scale.

Foam Symbol	Apparent Density, kg/m^3^	Closed-Cell Content, %	Thermal Conductivity Coefficient, mW/m∙K	LOI, % O_2_	Compressive Strength, kPa	Brittleness, %
DEG_t45_lab	12.23 ± 0.39	1.24 ± 1.68	36.96 ± 0.74	21.3	11.43 ± 0.26	2.73 ± 2.68
TEA_t45_lab	11.53 ± 0.11	2.21 ± 0.03	41.06 ± 0.39	21.5	12.86 ± 1.51	16.92 ± 6.20

**Table 6:** Properties of ocSPF obtained by spraying.

Foam Sample	Apparent Density, kg/m^3^	Closed-Cell Content, %	Thermal Conductivity Coefficient, mW/m∙K	LOI, % O_2_	Compressive Strength, kPa	Brittleness, %
DEG_p80_t52	16.22 ± 0.49	1.61 ± 1.40	37.06 ± 0.99	21.3	27.70 ± 1.45	4.32 ± 3.39
DEG_p90_t52	14.36 ± 0.88	2.35 ± 0.20	36.04 ± 0.89	21.3	13.30 ± 1.06	0.73 ± 0.34
DEG_p100_t52	14.16 ± 0.19	3.50 ± 1.24	35.95 ± 0.72	21.3	14.24 ± 1.18	0.69 ± 0.97
TEA_p80_t45	21.49 ± 0.13	4.17 ± 1.39	34.96 ± 1.03	21.5	43.82 ± 2.74	10.78 ± 1.57
TEA_p80_t52	17.59 ± 0.48	4.46 ± 0.07	36.59 ± 0.68	21.5	22.14 ± 3.37	8.92 ± 0.33
TEA_p90_t52	17.09 ± 0.14	4.85 ± 0.42	36.76 ± 1.20	21.5	26.88 ± 1.58	7.25 ± 3.79
TEA_p100_t52	17.38 ± 1.23	2.43 ± 0.03	36.62 ± 0.90	21.5	25.50 ± 2.00	7.39 ± 1.63

**Table 7:** Water-vapor permeability of ocSPFs.

Foam Sample	Water-Vapor Permeability δ, mg/(m*h*Pa)	Water-Vapor Diffusion Resistance Factor μ (Dimensionless)
DEG_p80_t52	0.27 ± 0.01	2.68 ± 0.13
DEG_p90_t52	0.24 ± 0.02	2.98 ± 0.29
DEG_p100_t52	0.14 ± 0.02	5.08 ± 0.55
TEA_p80_t45	0.21 ± 0.02	3.38 ± 0.38
TEA_p80_t52	0.24 ± 0.02	2.94 ± 0.19
TEA_p90_t52	0.25 ± 0.01	2.83 ± 0.12
TEA_p100_t52	0.26 ± 0.01	2.68 ± 0.09

**Table 8:** Morphology of PUR foams.

Foam Sample	Direction of Growth	Anisotropy Index	Cross-Section Area, mm^2^	Cell-Density × 10^3^, Number of Cells/cm^3^
DEG_p80_t52	Parallel	1.94 ± 0.54	0.071 ± 0.059	54.19 ± 0.65
	Perpendicular	1.44 ± 0.32	0.044 ± 0.038	
DEG_p90_t52	Parallel	1.66 ± 0.44	0.068 ± 0.052	62.51 ± 2.15
	Perpendicular	1.47 ± 0.41	0.039 ± 0.036	
DEG_p100_t52	Parallel	1.79 ± 0.58	0.049 ± 0.028	94.72 ± 0.11
	Perpendicular	1.50 ± 0.38	0.031 ± 0.020	
TEA_p80_t45	Parallel	2.02 ± 0.59	0.051 ± 0.040	124.23 ± 0.29
	Perpendicular	1.41 ± 0.31	0.021 ± 0.013	
TEA_p80_t52	Parallel	2.04 ± 0.67	0.082 ± 0.760	53.44 ± 2.85
	Perpendicular	1.54 ± 0.04	0.040 ± 0.036	
TEA_p90_t52	Parallel	1.91 ± 0.60	0.065 ± 0.057	63.74 ± 0.12
	Perpendicular	1.41 ± 0.32	0.038 ± 0.036	
TEA_p100_t52	Parallel	1.83 ± 0.55	0.078 ± 0.063	49.26 ± 1.13
	Perpendicular	1.48 ± 0.39	0.046 ± 0.042	

## Data Availability

The data presented in this study are available on request from the corresponding author. The data are not publicly available due to privacy concerns.
